# ICU without borders

**DOI:** 10.1186/s13054-023-04463-0

**Published:** 2023-05-13

**Authors:** Marlies Ostermann, Jean-Louis Vincent

**Affiliations:** 1 Department of Critical Care, King’s College London, Guy’s and St Thomas’ Foundation Hospital, NHS Foundation Trust, London, SE1 7EH UK; 2 Department of Intensive Care, Erasme University Hospital, Université Libre de Bruxelles, Route de Lennik 808, 1070 Brussels, Belgium

**Keywords:** Outreach, Follow-up, ICU without borders

## Abstract

Critical illness is a continuum, but patient care is often fragmented. Value-based critical care focuses on the overall health of the patient, not on an episode of care. The “ICU without borders” model incorporates a concept where members of the critical care team are involved in the management of patients from the onset of critical illness until recovery and beyond. In this paper, we summarise the potential benefits and challenges to patients, families, staff and the wider healthcare system and list some essential requirements, including a tight governance framework, advanced technologies, investment and trust. We also argue that “ICU without borders” should be viewed as a bi-directional model, allowing extended visiting hours, giving patients and families direct access to experienced critical care staff and offering mutual aid when needed.

## Background

**Fig. 1:**
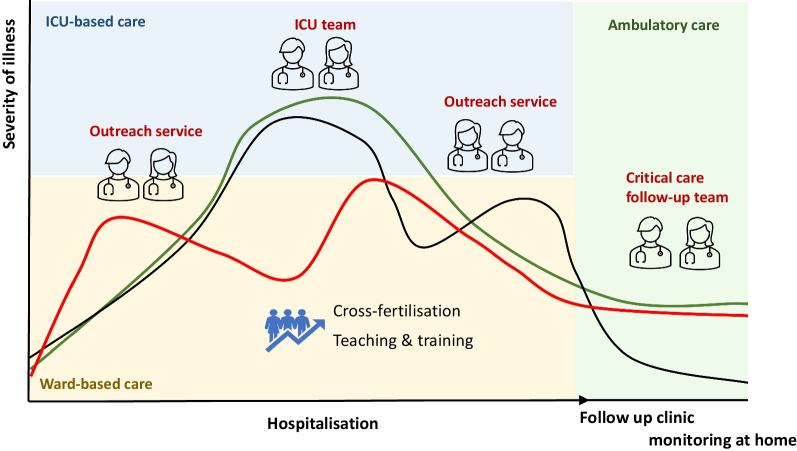
Critical illness as a continuum. ICU = intensive care unit

Critical illness is a dynamic continuum with various phases and trajectories, including deterioration, organ dysfunction and recovery or death. [1] (Fig. 1) Patient care, on the other hand, is traditionally fragmented where management is passed from one group of carers to another, usually within the same healthcare structure but occasionally between different organisations. At the interfaces between these groups, care often breaks down due to gaps in communication and collaboration between medical disciplines and staff groups. It is very likely that these gaps and delays impact patients’ outcomes. For instance, in one study of 401 general ward patients requiring admission to the Intensive Care Unit (ICU), each hour of delay in admission was associated with a 1.5% increase in the risk of ICU death and a 1% increase in hospital mortality. [2] Similarly, within the ICU, the critical care team is not always aware of all events and discussions prior to admission, and after patients leave the ICU, clinicians tend to lose sight of their progress unless readmission is necessary. ICU discharge summaries are often incomplete. [3, 4] Thus, vital information can get lost, opportunities for interventions may be delayed or missed, and errors occur. In this paper, we summarise the benefits and challenges of providing health care that focuses on the entire patient journey, and not on the individual episodes of care or location.

## Premises and opportunities

“ICU without borders” describes a concept where members of the critical care team are involved in the management of patients who are critically ill or deteriorating and at risk of becoming critically ill until recovery and even beyond. This model offers continuity of care with a streamlined transfer of vital information and coordinated care. Alerted by staff and/or electronic alerting systems, and supported by telemedicine where available, members of the critical care team can take part in the assessment and decision-making process from the moment patients are deteriorating outside of the ICU. [5] The team helps directing the next steps and participates in discussions about escalation of care. Appropriate care can then be initiated on the ward, from basic organ support to appropriate symptom relief and palliative care if needed. When successful, admission to ICU may be prevented or facilitated in a timely manner, depending on the circumstances. Additional benefits include education and training of ward staff and the relevant medical teams and the provision of emotional support to patients, families and clinical staff.

The transition from the ICU to the ward is a vulnerable time for patients, exposing them to anxiety and risk of adverse events, including ICU readmission and death. [6–8] The “ICU without borders” model provides a safety net and supports the continuing physical and emotional recovery of patients but also allows early identification of complications that may lead to another deterioration. Systematic reviews and meta-analyses have shown that ICU discharge follow-up programmes reduce the risk of ICU readmission. [9] Critical care support post ICU can also address the ward nurses’ anxiety associated with receiving ICU patients. [10]

The concept of “ICU without borders” can extend beyond hospital discharge. Post-ICU clinics run by members of the critical team provide patients and families opportunities to ask questions, raise concerns and fill gaps in their understanding. Wearable technologies and home-based rehabilitation programs have been shown to better identify and also ameliorate symptoms of post-intensive care syndrome. [11, 12] Finally, post-ICU follow-up clinics can offer care for family members who may be at risk of physical exhaustion, post-traumatic stress disorder, anxiety, and depression. [13]

In many institutions, these models have already been introduced successfully with effective outreach teams and ICU follow-up clinics, both led by critical care. New technologies and monitoring equipment exist that enable critical care teams to go further. For instance, it is possible to facilitate both pre-hospital treatment at home before and after hospitalisation and in transit. Technologies are available to better track and pass on patients’ preferences, such as advance directives and organ donation wishes. [11] It is very likely that future critical care will potentially include the patient’s home and field hospitals, similar to critical care telemedicine programs that support remote or low-resource environments with virtual input from experts around the world. [11]

“ICU without borders” should be regarded as a bi-directional model with “open doors” for patients, families and other health care providers. Extended (or open) family visiting hours enable contacts with the patient’s home life, friends and even visits of pets. [14] In some institutions, systems exist where patients and families on the wards are able to access the critical care team directly in case of concerns. [15, 16]

Finally, the COVID pandemic showed that a concept of “ICU without borders” allowed cross-border cooperation and mutual aid between institutions but also between regions with patients being transferred from one country to another. For example, patients from Denmark, France and Belgium were treated in ICUs in Germany, while Luxembourg provided ICU beds for Italian and French patients. During the early stages of the crisis, some member states also sent teams of doctors to their most severely impacted neighbours, providing critical countermeasures via the EU Civil Protection Mechanism.

## Potential challenges

To provide care that mirrors the continuum of critical illness, it is essential to have skilled staff and a flexible infrastructure with reliable alerting systems, tools for remote monitoring and opportunities to provide basic organ support when needed. Further, a tight governance structure with a clear chain of responsibility and accountability needs to be in place to avoid adverse incidents and conflict.

Having the critical care team involved throughout the continuum of critical care requires all members to acknowledge the skills and limitations of everybody whom they are working with. The model provides staff on the ward opportunities to be trained and to advance their skills but, on the other hand, it also increases the risk of de−skilling. A system that gives patients and families access to the medical records serves to empower them to contact the critical care team directly but may also lead to panicked calls and too many demands on the team. Further, critical care led follow−up clinics have been set up in many hospitals. However, a systematic review of four randomised controlled trials and one non−randomised study showed little to no evidence that they made any difference to all−cause mortality and quality of life at 12 months after ICU discharge.[17] The results of ongoing studies exploring the effectiveness of follow−up clinics are eagerly awaited. Finally, although cross−border cooperation is welcome in a crisis or pandemic, it is not without risk. ICU patients are by definition high−risk, with mortality rates often ranging between 20 to 50%. Transferring specialists rather than patients may be associated with more bureaucracy but less risks for patients.[18]

## Conclusions

**Fig. 2:**
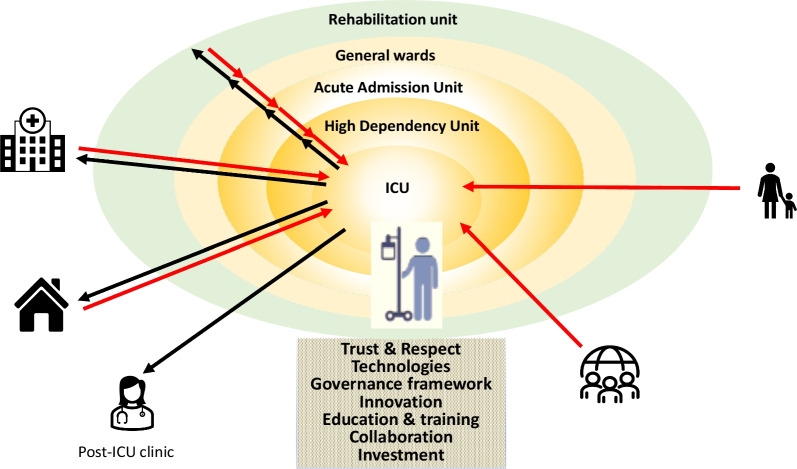
ICU without borders. ICU = intensive care unit

Critical illness needs to be seen as a continuous and dynamic sequence of interlinked events from the very early moments of illness, through the stay in hospital, including ICU, and into recovery and rehabilitation. (Fig. 1) Value-based critical care focuses on the outcomes that matter to the patient, independent of location or episode of care. Standardisation and consolidation (horizontal integration) and provision of care throughout the phases of critical illness with multi-professional collaboration (vertical integration) are needed. [19] The model of “ICU without borders” serves to achieve these goals, (Table 1, Fig. 2) It also offers potential for downstream cost-saving through early detection of patient deterioration, improved transfer of information and reduced waste of valuable resources (appointments for specialist investigations, for instance). Lastly, it provides great opportunities for multi-disciplinary research into all phases of critical illness. [13]

**Table 1:** Potential benefits and challenges of the “ICU without border” concept

Potential benefits	Challenges
Early recognition of deterioration	Need for sufficient number of staff
Prompt advice to the ward staff	Requires broad training program for critical care team
Timely admission to ICU if required	Risk of de-skilling of ward staff
Continuity of care	Need for tight governance structure and regulatory framework
Sharing of knowledge and cross-fertilisation	Model relies on trust, respect and collaboration between staff members
Potentially earlier discharge from ICU	Requires flexible infrastructure in non-critical areas
Support for patients discharged from ICU	Need for investment (telemedicine, training)
Support of ward staff receiving patients from ICU	Requirement for agreed admission and transfer criteria
Avoidance of unnecessary patient moves/transfers	Uncertainty about cost effectiveness
Less delays/waste of resources	
Opportunities for multidisciplinary research	
Mutual aid during a pandemic /natural disaster	
Preparedness for epidemics / disasters	
Support of patients at home (before and after hospitalisation)	

*ICU* intensive care unit

## Data Availability

Not applicable.

## Abbreviations

COVID: Coronavirus disease
ICU: Intensive Care Unit
